# Unusual report of an immunocompetent patient with atypical cutaneous leishmaniasis caused by *Leishmania (Leishmania) infantum* in Minas Gerais, Brazil

**DOI:** 10.1590/0037-8682-0314-2024

**Published:** 2025-09-29

**Authors:** Rebeca Mendes Rocha, Jackeline Maria de Sousa Lima Lopes, Emanuelle de Moura Santos Xavier, Rosanna Lorrane Francisco dos Reis Matos, Dayse Marcielle de Souza Lopes, Flávia Carvalho Bitencourt de Oliveira, Felipe Dutra Rêgo, José Dilermando Andrade, Célia Maria Ferreira Gontijo, Leandro de Freitas Teles, Jamille Fernandes Lula, Luciano Freitas Fernandes, André Luiz Sena Guimarães, Ana Paula Venuto Moura, Thallyta Maria Vieira, Sílvio Fernando Guimarães de Carvalho

**Affiliations:** 1 Universidade Estadual de Montes Claros, Programa de Pós-Graduação em Ciências da Saúde, Montes Claros, MG, Brasil.; 2 Centro de Referência em Doenças Infecciosas (CERDI) do Serviço de Atendimento Especializado (SAE) Ampliado, Secretaria Municipal de Saúde, Montes Claros, MG, Brasil.; 3 Universidade Estadual de Montes Claros, Hospital Universitário Clemente de Faria, Montes Claros, MG, Brasil.; 4 Fundação Oswaldo Cruz, Instituto René Rachou, Belo Horizonte, MG, Brasil.; 5 Hospital Santa Casa de Montes Claros, Montes Claros, MG, Brasil.; 6 Universidade Estadual de Montes Claros, Programa de Pós-Graduação em Modelagem Computacional e Sistemas, Montes Claros, MG, Brasil.; 7 Universidade Estadual de Montes Claros, Programa de Pós-Graduação em Biotecnologia, Montes Claros, MG, Brasil.; 8 Universidade Estadual de Montes Claros, Departamento de Biologia Geral, Montes Claros, MG, Brasil.; 9 Universidade Estadual de Montes Claros, Programa de Pós-Graduação em Biodiversidade e Uso dos Recursos Naturais, Montes Claros, MG, Brasil.; 10 Universidade Estadual de Montes Claros, Programa de Pós-Graduação em Cuidado Primário em Saúde, Montes Claros, MG, Brasil.

**Keywords:** Cutaneous leishmaniasis, Leishmania infantum, Northern Minas Gerais

## Abstract

Cutaneous leishmaniasis is endemic in Minas Gerais, Brazil; however, data on the circulating species remain limited. This study identified *Leishmania* species in he skin samples of patients in Montes Claros using molecular methods. *Leishmania (Viannia) braziliensis* was found in ten samples, and *Leishmania (Leishmania) infantum* in one, representing an unusual case of cutaneous leishmaniasis caused by *L. infantum* in an immunocompetent patient in the state. The patient, a 62-year-old woman presented with four atypical papular lesions on the arm, forearm, and back, with no systemic symptoms. These results highlighted the need for enhanced epidemiological surveillance.

## INTRODUCTION

Cutaneous leishmaniasis (CL) is a non-contagious infectious disease caused by intracellular protozoa of the *Leishmania* genus that is transmitted mainly through the blood meal of infected female phlebotomine sandflies^1^.

In the Old World, the *Leishmania* species responsible for CL differ from those predominant in the New World, with *Leishmania (Leishmania) infantum* emerging as a significant etiological agent of CL transmission^2^. In the New World, this species is classically associated with visceral leishmaniasis (VL); cutaneous cases of VL are rare and poorly documented^1^ ^,^ ^3^.

Recent evidence indicates that *L. (L.) infantum* can cause cutaneous manifestations in different geographic settings, including Central America, where clinical, parasitological, and immunological analyses have identified this species as the etiological agent of non-ulcerated or atypical cutaneous lesions^4^.

In Brazil, research using databases such as PubMed and SciELO has revealed case reports from various regions, including Rio de Janeiro^5^, Mato Grosso do Sul^6^, the interior of São Paulo state^7^, and, more recently, the Northeast region^8^. The identification of *L. (L.) infantum* in nonulcerated or atypical cutaneous lesions suggests diversity in the tropism and virulence of the species, emphasizing the importance of molecular characterization for accurate diagnosis and epidemiological surveillance^9^ ^,^ ^10^. The absence of species-level identification in the routine diagnosis of CL likely results in a significant underestimation of the true number of cases.

In Minas Gerais (MG), CL is endemic, with the northern mesoregion being a high incidence area for the disease¹¹. Despite the increasing number of cases, studies identifying the *Leishmania* species circulating in the region remain scarce. This case report describes the identification of Leishmania species in patients with CL in Montes Claros, MG, Brazil, and reports an unusual exclusively cutaneous case in an immunocompetent human, caused by *L. (L.) infantum* in an atypical papular lesion in the state.

## CASE REPORT

**FIGURE 1:**
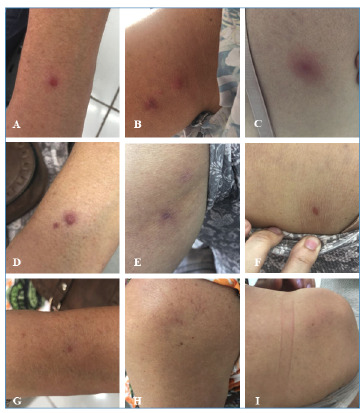
Clinical progression of a 62-year-old female patient with cutaneous leishmaniasis caused by *Leishmania (Leishmania) infantum* (accession KT966382.1), presenting four localized papular lesions: one on the right side of the back, two on the left arm, and one on the left forearm. **Images A-C:** lesions before treatment initiation. **Images D-F:** images taken after treatment with miltefosine, on the day of the first intralesional meglumine antimoniate session. **Images G-I:** appearance of the lesions at medical discharge, following complete resolution.

Among the 19 patients with a confirmed diagnosis of CL treated at the Center for Reference in Infectious Diseases of Montes Claros, MG (CERDI), one presented an atypical profile in terms of the identified species and the clinical course, standing out from the others and forming the focus of this report. The patient was a 62-year-old woman who lived in an urban area of Montes Claros, MG. She was retired and had a medical history of systemic arterial hypertension, anxiety disorder, and insomnia, and was under continuous use of Nebilet® (Nebivolol Hydrochloride), Xarelto® (Rivaroxaban), and Alprazolam® (Alprazolam). She presented with four non-ulcerated papular lesions, approximately one centimeter in diameter, located on the back (one lesion on the right side), upper arm (two lesions on the left side), and forearm (one on the left side), with an estimated duration of 12 months (Figure 1 (A-C). Before seeking specialized care, she used over-the-counter ointments without any therapeutic success.

At the time of consultation at the CERDI, the patient had already undergone a histopathological examination consistent with tegumentary leishmaniasis, indicating chronic ulcerated granulomatous dermatitis. A second biopsy was performed at the inner edge of the lesion for diagnostic confirmation using molecular methods. The samples were collected by a specialist physician and sent for DNA extraction using a High Pure PCR kit (Roche Diagnostics GmbH) following the manufacturer’s instructions. Subsequently, amplification of the ITS1 region (~300-350 bp), a non-coding segment of the *Leishmania* genome, was performed using the primers LITSR (5' CTGGATCATTTTCATG 3') and L5.8S (5' TGATACCACTTATCGCACTT 3').

This procedure was applied to all 19 samples included in the study. *Leishmania* DNA was detected in 14 of these samples and was subjected to Restriction Fragment Length Polymorphism (RFLP) analysis using the restriction enzyme *HaeIII*. Positive controls included DNA from *L. (V.) braziliensis*, *L. (L.) amazonensis*, *L. (V.) guyanensis*, and *L. (L.) infantum*, and a lesion sample not compatible with cutaneous leishmaniasis was used as a negative control.

The sample from the patient showed a typical digestion pattern consistent with *L. (L.) infantum*, which prompted genetic sequencing using the Sanger method. After purification with the Illustra™ GFX PCR and Gel Band Purification kit (GE Healthcare), sequence alignment revealed 100% identity with *L. (L.) infantum* (KT966382.1). The patient underwent a rapid test for visceral leishmaniasis (VL) and laboratory tests for liver function, which were negative; the laboratory results showed no signs indicative of visceral involvement.

Treatment was initiated with miltefosine (50 mg, three times daily for 28 days), which resulted in complete healing of three of the four lesions. However, the lesion on the left forearm showed no signs of regression, even after completion of the treatment regimen (Figure 1 (D-F) . Due to partial treatment failure, rescue therapy was initiated four months after the completion of oral treatment. The patient received intralesional injections of meglumine antimoniate (approximately 1 mL) administered in three consecutive biweekly sessions, leading to complete resolution of the remaining lesion (Figure 1 (G-I) . The patient was followed up for 12 months without any signs of relapse or progression to visceral leishmaniasis.

As part of the study, the other patients (n=18) with confirmed cutaneous leishmaniasis were also subjected to the same diagnostic protocol. Among the 14 cases with detected DNA, 12 showed profiles consistent with *L. (Viannia) braziliensis* after RFLP analysis, which was later confirmed by sequencing (similarity ranging from 97.41% to 100% with MT606274.1 and MT940880.1). Two samples were identified as *Leishmania* spp. because of poor sequence quality, and one result was inconclusive. In the case of *L. (V.) braziliensis*, most patients were male (n=6; 60%), aged between 34 and 77 years, and predominantly resided in urban areas (n=7; 70%). The clinical presentations included localized (n=6; 60%), disseminated (n=2; 20%), and mucocutaneous (n = 2; 20 %) forms.

## DISCUSSION

*L. (V.) braziliensis* was the most frequently identified species in our study, likely because it is responsible for the majority of cutaneous leishmaniasis cases in Brazil^1^ ^,^ ^11^. In a study conducted by Quaresma et al.^12^, a variant of this species was described in lesion samples from patients in the Xakriabá Indigenous Territory, which is also located in northern Minas Gerais.

The municipality of Montes Claros is also endemic for visceral leishmaniasis^3^, indicating the presence of *L. (L.) infantum* in the region. Although the presence of this species in cutaneous lesions is uncommon, the lack of studies aimed at identifying the species involved in cutaneous leishmaniasis cases in this area has contributed to the scarcity of studies on previously identified species.

In a study by Maia et al.^9^, comparing the experimental transmission to mice of a viscerotropic strain (IMT373) and a dermotropic strain (CUK3) of *L. (L.) infantum*, using natural vector species, it was observed that the parasite load transmitted by female *Lutzomyia longipalpis* was higher for the dermotropic strain. The presence of the parasite in the cutaneous tissues can elicit a localized immune response that is capable of causing skin damage.

It is believed that the inherent characteristics of the infecting species, in association with the host immune status and vector-related factors, largely determine the tropism of cutaneous leishmaniasis. Additionally, dissemination of the etiological agent may depend on the inoculum size and the specific ability of *Leishmania* species to become visceral^10^.

In conclusion, this study identified *Leishmania* species involved in cases of cutaneous leishmaniasis and, for the first time, characterized *Leishmania (Leishmania) infantum* as the causative agent of cutaneous leishmaniasis in humans in Montes Claros, Minas Gerais, Brazil. This finding enhances our understanding of the disease in this region and suggests the need for further investigation into the specific characterization of the etiological agent in human cases. Cutaneous leishmaniasis caused by *Leishmania (Leishmania) infantum* may not be an isolated occurrence; it represents a new challenge for the diagnosis, treatment, prevention, and control of leishmaniasis in this region.

## Data Availability

Data available upon request.
